# A Novel Signature of Lipid Metabolism-Related Gene Predicts Prognosis and Response to Immunotherapy in Lung Adenocarcinoma

**DOI:** 10.3389/fcell.2022.730132

**Published:** 2022-02-28

**Authors:** Kai Zhang, Ying Qian, Xiaowei Quan, Tengteng Zhu, Biyun Qian

**Affiliations:** ^1^ Shanghai Tongren Hospital and Faculty of Public Health, Hongqiao International Institute of Medicine, Shanghai Jiao Tong University School of Medicine, Shanghai, China; ^2^ Hongqiao International Institute of Medicine, Shanghai Tongren Hospital and Clinical Research Institute, Shanghai Jiao Tong University School of Medicine, Shanghai, China; ^3^ Shanghai Hospital Development Center, Shanghai, China

**Keywords:** lipid metabolism, lung adenocarcinoma, tumor microenvironment, immunotherapy, prognosis model, immune checkpoint

## Abstract

**Background:** Lipid metabolism disorder, a new hallmark of cancer initiation, has been involved in lung adenocarcinoma (LUAD). However, few biomarkers about lipid metabolism-related genes (LMRGs) have been developed for prognosis prediction and clinical treatment of LUAD patients.

**Methods:** In this study, we constructed and validated an effective prognostic prediction model for LUAD patients depending on LMRGs. Subsequently, we investigated the prediction model from immune microenvironment, genomic changes, and immunotherapy.

**Results:** Then, eleven LMRGs were identified and applied to LUAD subtyping. In comparison with the high-risk group, the low-risk group exhibited a remarkably favorable prognosis, along with a higher immune score and lower tumor purity. Moreover, the low-risk group presented higher levels of immune checkpoint molecules, lower tumor immune dysfunction and exclusion (TIDE) score and tumor mutation burden (TMB), and higher likelihood of benefiting from immunotherapy. Furthermore, the genomic changes of six LMRGs (CD79A, HACD1, CYP17A1, SLCO1B3, ANGPTL4, and LDHA) were responsible for the difference in susceptibility to LUAD by greatly influencing B-cell activation.

**Conclusion:** Generally speaking, the LMRG model is a reliable independent biomarker for predicting adverse outcomes in LUAD patients and has the potential to facilitate risk-stratified immunotherapy.

## Introduction

Lung cancer, composed of approximately 85% non-small-cell lung cancer (NSCLC) and 15% small cell lung cancer (SCLC), is one of the most prevalent malignant cancers worldwide, with over 1.4 million deaths each year (Zarogoulidis et al., 2013). To date, lung adenocarcinoma (LUAD) has exceeded lung squamous cell carcinoma (LUSC) in the morbidity and become the most common pathological type of lung cancer (Bray et al., 2018). As we all know, diagnosed at an advanced stage remains a major cause of the high mortality of LUAD. Traditional prognostic prediction still relies on histopathologic diagnosis and tumor stage. However, these models fail to identify high-risk population and predict LUAD patients who are more likely to benefit from immunotherapies. Hence, it is imperative to explore accurate and effective prognostic biomarkers and models to assist clinical individualized treatment.

Metabolic reprogramming, a well-established hallmark of cancer, is critical for tumor initiation and progression (Hanahan and Weinberg, 2011). Lipids are composed of thousands of different molecules, including phospholipids, fatty acids, triglycerides, and sphingolipids. Fatty acids and cholesterol are the basic structure of cell membrane, which play a significant role in tumor cell proliferation, invasion, and metastasis (Orita et al., 2008). Lipids also can act as second messengers to transmit signals in tumor cells and participate in energy supply to tumor progression (Orita et al., 2007). In recent years, plenty of studies have focused on revealing the molecular mechanisms and signal pathways of LUAD caused by alteration of lipid composition. Hall et al. confirmed that MYC expression drives aberrant lipid metabolism in LUAD using the transgenic mouse model (Hall et al., 2016). Masri et al. found that the STAT3–SOCS3 pathway mediated the changes of insulin, glucose, and lipid metabolism in lung adenocarcinoma-bearing mice (Masri et al., 2016). These findings confirm the role of lipid metabolism in LUAD and indicate that lipid metabolism-related genes (LMRGs) may have a great prospect to act as prognostic markers of LUAD.

Therefore, we integrated gene expression and survival information of LUAD patients from TCGA and GEO databases to construct and validate a gene signature related to lipid metabolism to assess the risk of adverse outcomes. We then used this gene signature to divide LUAD patients into high- and low-risk groups and analyze the differences of signaling pathways and tumor microenvironment. On this basis, we identified the potential molecule affecting the response to immunotherapy in LUAD patients. Taken together, our study aimed to construct a robust prognosis prediction model based on LMRGs and provide an effective tool for immunotherapy of LUAD patients.

## Materials and Methods

### Patient Data Collection and Procession

The level 3 RNA sequencing profile and matching clinical data of 500 LUAD tumor tissues and 59 adjacent non-tumorous tissues in The Cancer Genome Atlas (TCGA) were downloaded using the R package “TCGAbiolinks,” whose expression profiles were normalized using the R package “Deseq2.” RNA-seq expression matrix and clinical data of GSE31210 (226 lung adenocarcinoma samples; GPL570 platform) and GSE3141 (111 lung adenocarcinoma samples; GPL570 platform) were obtained from the Gene Expression Omnibus (GEO) database using the R package “GEOquery,” whose expression profiles were normalized using the R package “limma” and served as the validation set. The data from TCGA and GEO are both publicly available and were used following the TCGA and GEO data access policies and publication guidelines. Eleven cases, with matched non-tumor and LUAD tumor tissue samples from the Shanghai Tongren Hospital of Shanghai Jiaotong University, were enrolled in this study. The CD79A mRNA expression profiling of GSE11969 (90 tumor samples and 5 non-tumor samples of lung adenocarcinoma; GPL7015 platform), GSE30219 (85 tumor samples and 14 non-tumor samples of lung adenocarcinoma; GPL570 platform), GSE31210 (226 tumor samples and 20 non-tumor samples of lung adenocarcinoma; GPL570 platform), GSE32683 (53 lung adenocarcinomas and 53 matched adjacent non-malignant lung samples; GPL570 platform), GSE75037 (83 lung adenocarcinomas and 83 matched adjacent non-malignant lung samples; GPL6884 platform), and GSE81089 (108 tumor samples and 19 non-tumor samples of lung adenocarcinoma; GPL570 platform) was downloaded from the GEO database and compared between tumor and non-tumor tissues of LUAD patients with “limma” R package. A total of 500 samples from the TCGA-LUAD cohort were divided into high- and low-risk groups by the risk prediction model. Then, the “limma” R package was utilized to analyze the mRNA expression profile between two groups to screen out DEGs with a threshold of FDR < 0.05.

### Identification of LMRGs for Prognostic Prediction

Five lipid metabolism-related gene sets, including Hallmark fatty acid metabolism, KEGG glycerophospholipid metabolism, lipid raft, Reactome metabolism of lipids and lipoproteins, and Reactome phospholipid metabolism, were selected and extracted from Molecular Signatures Database v 7.2 (MSigDB) (Subramanian et al., 2005). After removing the overlapped genes, 664 lipid metabolism-related genes were acquired.

Differentially expressed genes between tumor samples and normal samples in TCGA-LUAD cohort were analyzed with the R package “DESeq2” based on the mRNA expression data of raw counts. A threshold of FDR less than 0.05 (FDR < 0.05) and the absolute log_2_ fold-change greater than 1 (|log_2_FC| > 1) were set to define differentially expressed genes (DEGs). Finally, 217 LMRGs were obtained by intersecting DEGs and genes involved in five lipid metabolism-related gene sets.

### Construction and Evaluation of the Prognostic Signature

First, univariate Cox regression analysis of overall survival (OS) was conducted to prefilter lipid metabolism-related genes (LRGs) with prognostic values. The least absolute shrinkage and selection operator (LASSO) Cox regression analysis was used to narrow the range of variables with the R package “glmnet.” Multivariate Cox stepwise regression analysis was applied to ultimately determine the target variable. Then, an LRG-related prognostic gene signature was constructed based on a linear combination of the regression coefficient derived from the multivariate Cox stepwise regression model coefficients (β) multiplied with its mRNA expression level. The detailed calculation formula was presented as follows: risk score = $\overset{n}{\underset{i=1}{\sum }}coefi\;x\;Genei$ , where “coefi” and “Genei” denote the coefficient and mRNA expression levels of each lipid metabolism-related gene. All patients were stratified into high-risk and low-risk groups based on the median value of the calculated risk score. According to the expression profile of selected genes in the signature, PCA and t-SNE were conducted to explore the distribution of high- and low-groups with R packages “ggfortify” and “Rtsne,” respectively. The Kaplan–Meier survival curve combined with a two-tailed log-rank test was used to compare the survival probability between the high- and low-risk groups by R packages “survival” and “survminer.” The time-dependent receiver-operating characteristic (ROC) curve was applied to evaluate the predictive power of the gene signature for OS by the R package “survivalROC.” The prognostic model was also validated in two independent cohorts from GSE31210 and GSE3141.

### Establishment and Evaluation of the Nomograms for LUAD Survival Prediction

Univariate and multivariate Cox regression analyses were utilized to identify whether the risk score and other clinicopathological variables (including age, gender, pathological stage, tumor stage, and risk score) could be independent of this prognostic model for LUAD patients. Subsequently, we employed all independent clinical prognostic factors selected from previous analysis to construct a nomogram which can assess the OS probability of 1, 2, and 3 years in LUAD patients. The 1-year, 2-year, and 3-year calibration curves were drawn to compare the observed prediction probability with the actual OS probability to verify the accuracy of the nomogram. Overlapping the reference line showed the great accuracy of the model. Decision curve analysis (DCA) curves were drawn to visually evaluate the net benefit for different independent clinical prognostic factors of the model.

### Function Enrichment Analysis

Risk score-related DEGs (|log_2_FC| ≥ 1, FDR < 0.05) were identified between high- and low-risk groups. Then, Gene Ontology (GO) and gene set enrichment analysis (GSEA) were conducted to explore enriched terms predicted to have an association with the Kyoto Encyclopedia of Genes and Genomes (KEGG) pathway in all hallmarks with R packages “clusterProfiler” and “GSEABase,” respectively.

### Immunohistochemical (IHC) Analysis

Immunohistochemically stained images were downloaded from the Human Protein Atlas (HPA) (https://www.proteinatlas.org/) and compared between tumor and non-tumor tissues of LUAD patients.

### Mutant Gene Analysis

TCGA-LUAD mutation data with the non-synonymous mutations and non-sense mutations included were downloaded with “TCGAbiolinks” package and analyzed and visualized using “maftools” package. The corresponding TMB value of each sample was obtained by calculating the mutations per million bases.

### Tumor Immune Dysfunction and Exclusion (TIDE) Analysis

TIDE is an effective algorithm that integrates two mechanisms of tumor immune escape—T-cell dysfunction and T-cell exclusion, which could be useful to predict immune checkpoint inhibitor (ICI) response in cancer treatment. The TIDE online web (http://tide.dfci.harvard.edu) was used to calculate the TIDE score, MSI score, T-cell exclusion score, and T-cell dysfunction score with the normalized RNA-seq data.

### Copy Number Variation (CNV) Analysis

After TCGA-LUAD CNV data were downloaded with “TCGAbiolinks” package and combined with the mRNA expression data, the local Perl script was used to calculate the amplification and deletion frequency of each sample. The location of 11 hub genes was identified and visualized with “RCircos” R package.

Evaluation of Tumor-Infiltrating Immune Cells (TIICs) and Immune Checkpoint Inhibitors (ICIs) Using ssGSEA, CIBERSORT, and ESTIMATE Algorithms and TIMER and TISIDB Databases

Single-sample gene set enrichment (ssGSEA) analysis was conducted to calculated the TIIC abundance profiles in LUAD tumor samples with “GSVA” package. The CIBERSORT algorithm was used to quantify the abundance of 22 immune cell type signature (LM22) subtypes, including seven T-cell types, two NK-cell types, three macrophage cell types, two B-cell types, two dendritic cell types, two mast cell types, plasma cells, monocytes, eosinophils, and neutrophils, in both high- and low-risk groups. The ESTIMATE algorithm was utilized to estimate the ratio of the immune–stromal components in the TME for each sample calculated with the immune score, stromal score, and ESTIMATE score with “estimate” R package. The “Gene” module of TIMER (https://cistrome.shinyapps.io/timer/) and TISIDB (http://cis.hku.hk/TISIDB/index.php) was used to explore the association of abundance of tumor-infiltrating lymphocytes (TILs) with gene expression. In addition, the mRNA level of 25 critical immune checkpoints obtained from the literature between high- and low-risk groups was compared in TCGA-LUAD cohort.

### Statistical Analysis

All data were presented as mean ± SD and analyzed with R studio software (Version 1.2.5001). An unpaired two-tailed Student’s t-test between two groups was performed. The Kaplan–Meier plot and log-rank test were applied to show the survival difference between high- and low-risk groups with R package “Survival.” Harrell’s concordance index (C-index) was computed for the predictive accuracy of the prognostic model. A *p* value <0.05 was considered statistically significant.

## Results

### Identification of Prognostic LMRGs in the TCGA-LUAD Cohort

**FIGURE 1:**
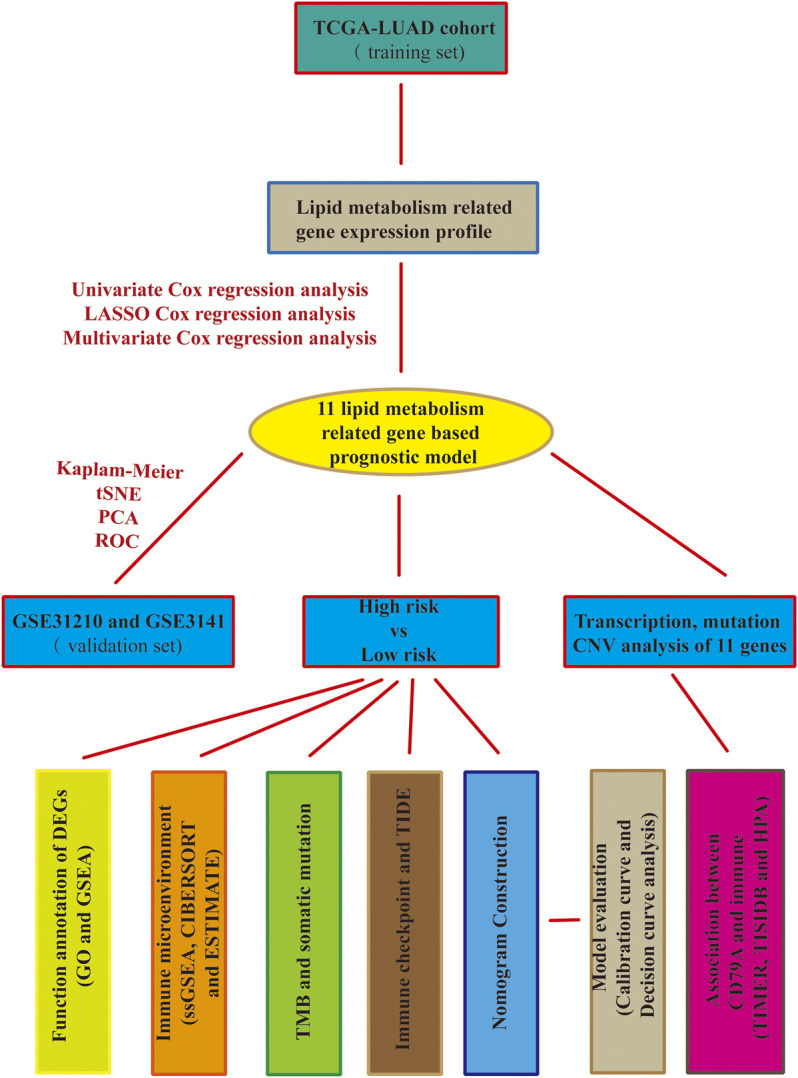
Flow chart of this study.

**FIGURE 2:**
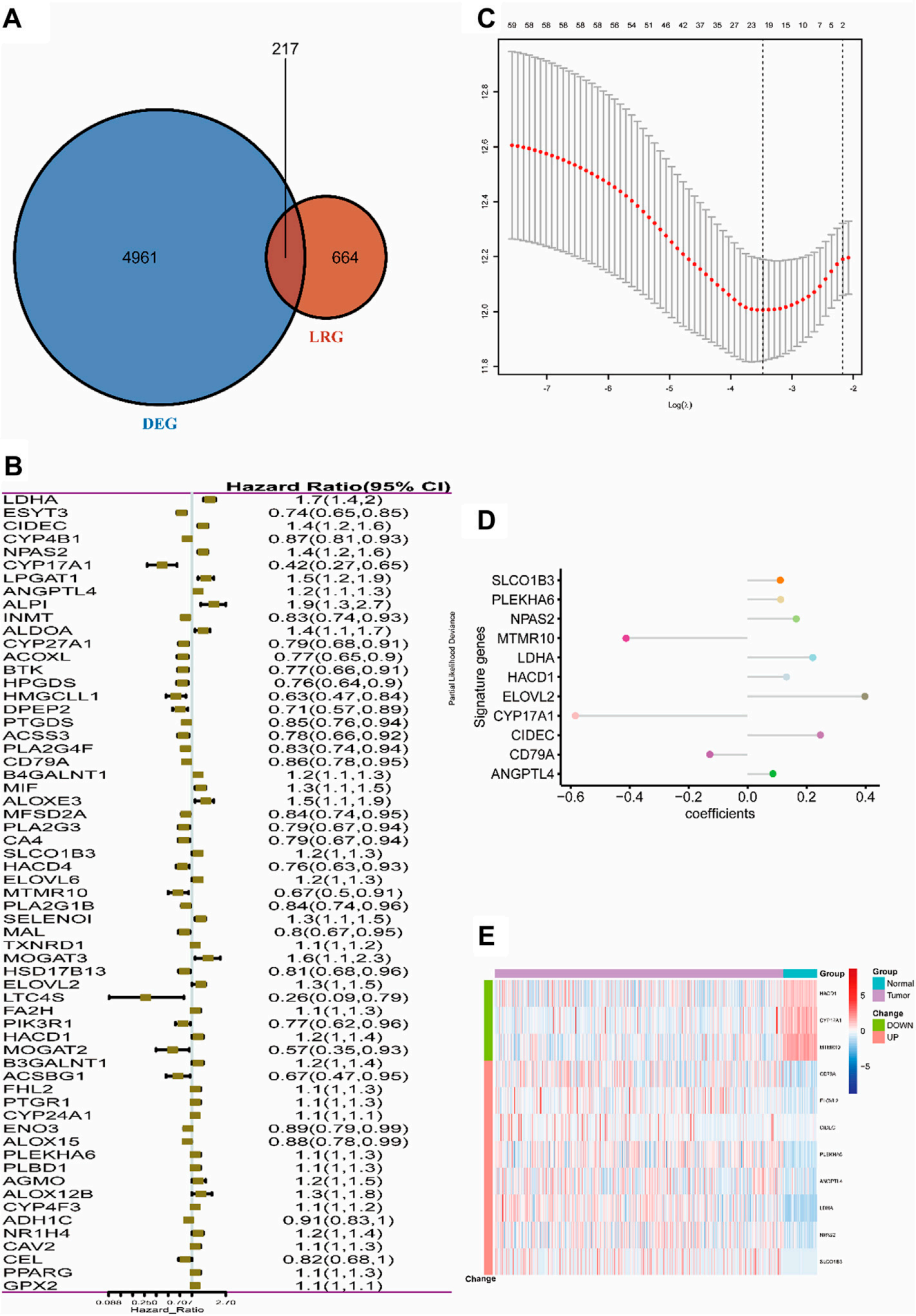
Selection of LMRGs associated with the survival of lung adenocarcinoma. **(A)** Venn diagram shows the intersection of DEGs and LRGs. **(B)** Forest plot of LMRGs associated with TCGA-LUAD survival. **(C)** Selection of the optimal parameter (lambda) in the LASSO Cox model for TCGA-LUAD. **(D)** LASSO coefficients of the 11 hub genes in TCGA-LUAD. **(E)** Heatmap of top 11 hub genes in TCGA-LUAD. “Group” means the tumor or adjacent tissues of TCGA-LUAD samples, while “Change” represents the expression trend of 11 lipid metabolism-related genes in tumor tissues when compared with adjacent tissues. DEG, differentially expressed gene; LMRG, lipid metabolism-related gene. Construction of the 11-LRG-based prognostic model in the TCGA-LUAD cohort.

The entire work of this study was conducted as described in the flow chart (Figure 1). A total of 217 (32.7%) LMRGs (Figure 2A) were differentially expressed between tumor tissues (*n* = 500) and adjacent tissues (*n* = 59), 61 of which were remarkably associated with overall survival (OS) with the univariate Cox regression analysis (Figure 2B).

**FIGURE 3:**
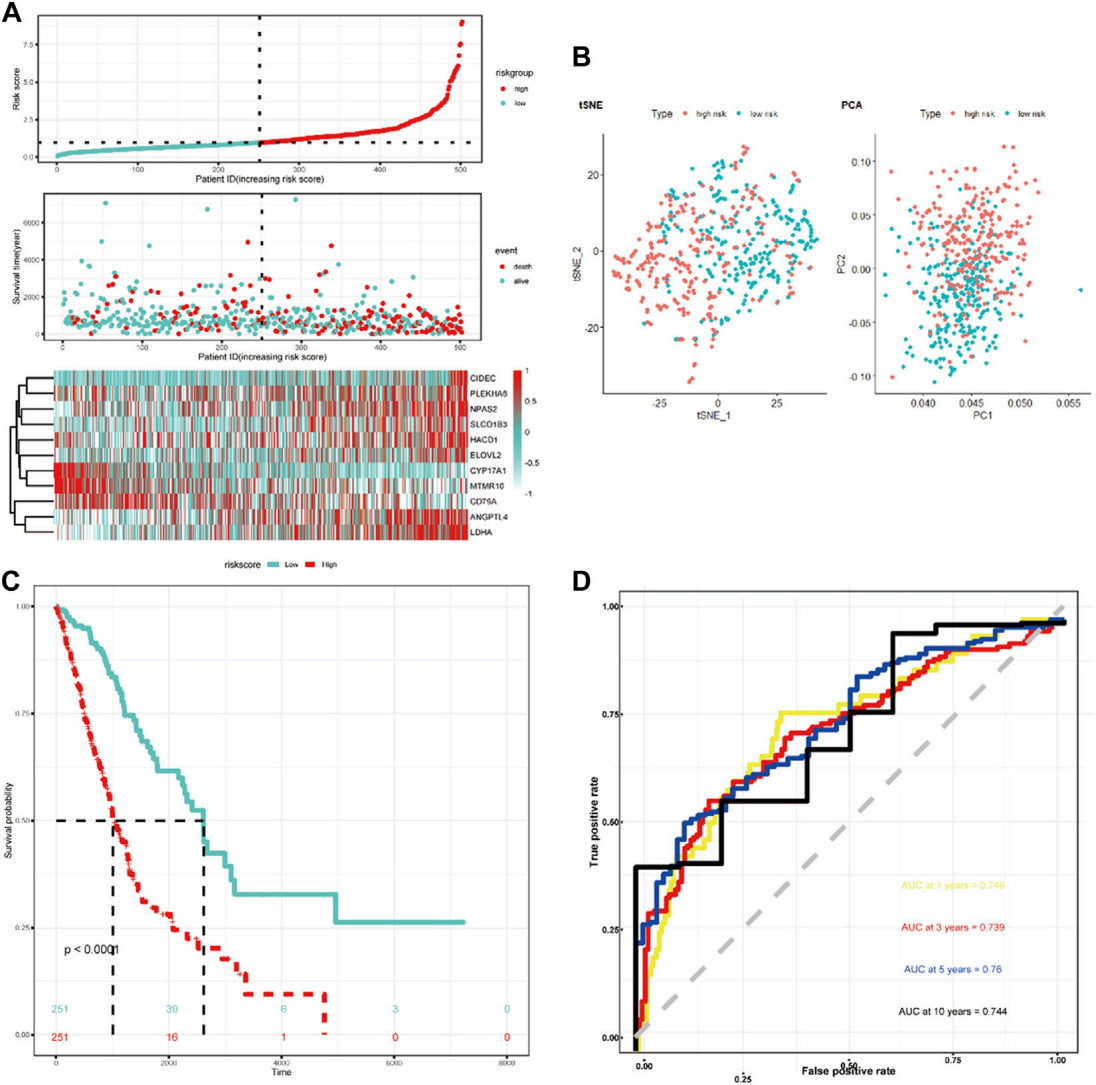
Characteristics of the LRG-related prognostic gene signature. **(A)** Heatmap of the 11-LRG-related gene expression profiles and the distribution of risk score and survival time/status for TCGA-LUAD. **(B)** t-SNE and PCA plot of 11-LRG-related gene expression profiles. **(C)** The Kaplan–Meier curve was plotted to estimate the overall survival probabilities of the low- and high-risk groups. **(D)** The ROC curve was plotted to predict the 1-, 3-, 5-, and 10-year prognosis.

LASSO Cox regression analysis was employed to narrow the genes to 20 from 61 genes mentioned above (Figure 2C). Finally, an 11-LRG-based signature including ANGPTL4, CD79A, CIDEC, CYP17A1, ELOVL2, HACD1, LDHA, MTMR10, NPAS2, PLEKHA6, and SLCO1B3 was identified with multivariate stepwise regression analysis and subsequently used to construct a clinical prognostic model. The risk score of each LUAD patient was calculated with the coefficients (Figure 2D) achieved from the multivariate stepwise regression analysis. The detailed calculation formula was presented as follows: 0.086 × expression level of ANGPTL4 + (−0.128) × expression level of CD79A + 0.247 × expression level of CIDEC + (−0.584) × expression level of CYP17A1 + 0.398 × expression level of ELOVL2 + 0.132 × expression level of HACD1 + 0.221 × expression level of LDHA + (−0.412) × expression level of MTMR10 + 0.165 × expression level of NPAS2 + 0.112 × expression level of PLEKHA6 + 0.111 × expression level of SLCO1B3. Compared with normal tissues, three genes (CYP17A1, HACD1, and MTMR10) were down-regulated in tumor tissues, while the others were up-regulated (Figure 2E). Then, all TCGA-LUAD patients were stratified into a high-risk group (*n* = 250) and a low-risk group (*n* = 250) according to the median cut-off value (Figure 3A). Patients in the high-risk group had a higher probability of premature death compared with those in the low-risk group. t-SNE and PCA showed that the 11-gene signature expression of patients in the high- and low-risk groups was clearly divided into two clusters (Figure 3B). Consistently, dramatically worse OS was observed in the high-risk group than the low-risk group through the Kaplan–Meier plot (*p* < 0.0001) (Figure 3C). After that, the time-dependent ROC curve was utilized to evaluate the prediction performance of the risk score. It was found the predicted area under the curve (AUC) of this model for 1-year, 3-year, 5-year, and 10-year overall survival rates reached 0.749, 0.739, 0.76, and 0.744, respectively (Figure 3D). The C-index for this clinical prognostic model reached 0.735.

**FIGURE 4:**
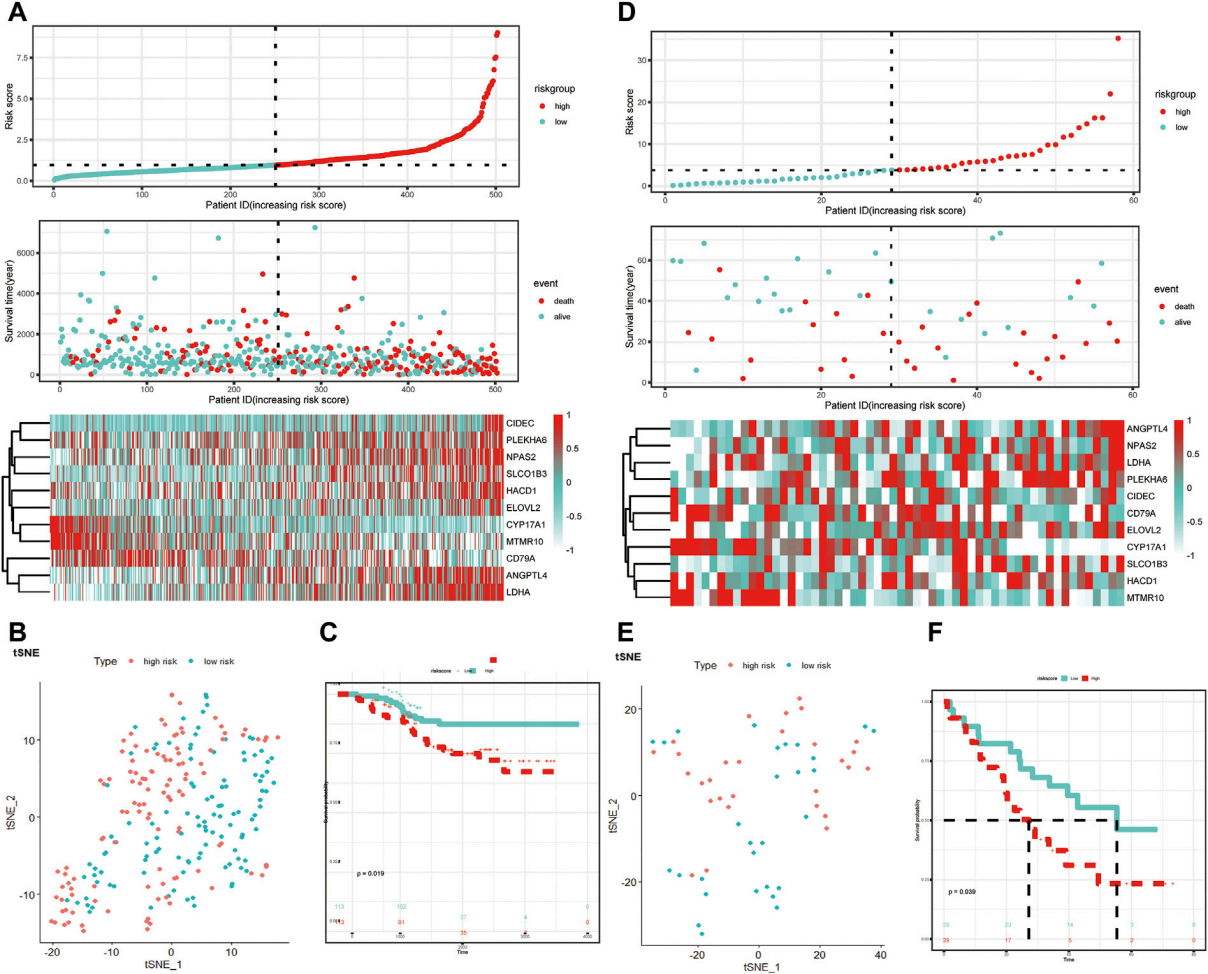
Validation of the LRG-related prognostic model. **(A,D)** Heatmap of the 11-LRG-related gene expression profiles and the distribution of risk score and survival time/status for GSE31210 and GSE3141. **(B,E)** t-SNE and PCA plot of 11-LRG-related gene expression profiles for GSE31210 and GSE3141. **(C,F)** The Kaplan–Meier curve was plotted to estimate the overall survival probabilities of the low- and high-risk groups for GSE31210 and GSE3141. Diverse tumor immune microenvironment between high- and low-risk LUAD patients.

The GSE31210 and GSE3141 external validation cohort was utilized to verify the robustness of the 11-LRG-based risk model. We calculated the risk score of two validation datasets with the above formula, based on the median using which patients were divided into high-risk and low-risk groups, respectively. The results showed patients with high risk score had worse OS in both GSE31210 and GSE3141, which was in line with that of TCGA-LUAD cohort (Figures 4A,D). In addition, t-SNE dimensionality reduction analysis found the mRNA profiles of 11 genes of the high- and low-risk groups were distinctly divided into two clusters in both GSE31210 and GSE3141 (Figures 4B,E). Furthermore, KM curves demonstrated patients in the low-risk group had higher survival probability than those in the high-risk group in both GSE31210 (*p* = 0.019) and GSE3141 (*p* = 0.039) (Figures 4C,F).

**FIGURE 5:**
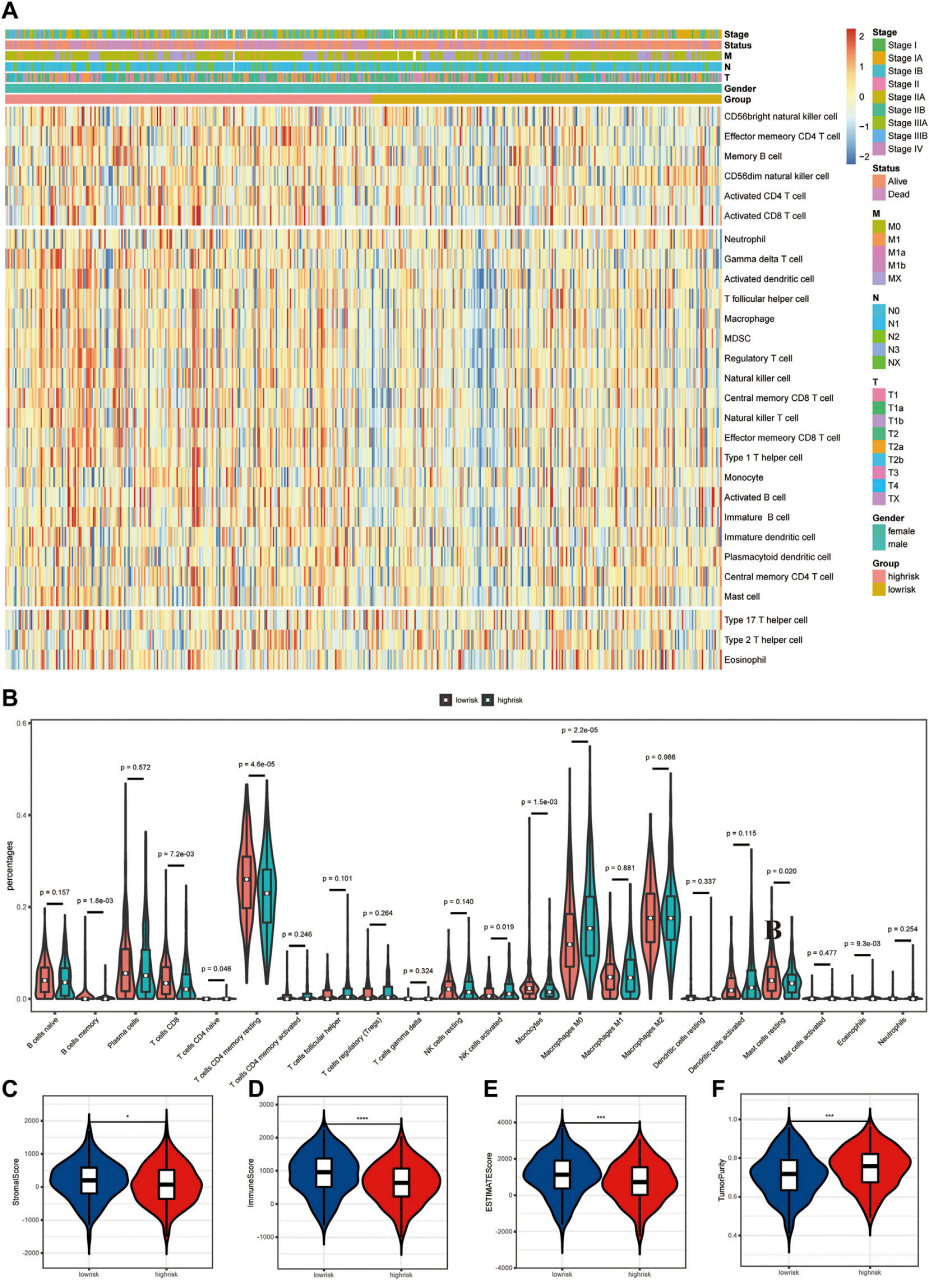
Differences in tumor immune microenvironment (TME) between high- and low-risk LUAD patients. **(A)** Heatmap of 28 infiltrating immune cell levels between high and low risks with ssGSEA. **(B)** Comparisons of 22 important immune fractions between high and low risks with CIBERSORT. **(C–F)** Comparison of the ESTIMATE score, stromal score, immune score, and tumor purity between high and low risks with ESTIMATE.

Differences in tumor immune microenvironment were explored to distinguish high- and low-risk LUAD patients. The ssGSEA results showed that the infiltrating levels of neutrophils, aDCs, iDCs, pDCs, macrophages, γδT, Tfh, Th1 cells, Tregs, NK cells, and B cells were notably elevated with the increased risk score (Figure 5A), indicating the activation of the body’s immune response. In order to further clarify the immune cell infiltration status between the high- and low-risk groups, the infiltration of 22 kinds of tumor-infiltrating immune cells (TIICs) was analyzed with the CIBERSORT algorithm. Compared with TIICs of LUAD patients in the low-risk group, B-cell memory (*p* = 0.0018), T-cell CD8 (*p* = 0.0072), T-cell CD4 memory resting (*p* < 0.0001), monocytes (*p* = 0.0015), and mast cell resting (*p* = 0.020) remarkably decreased, while T-cell CD4 naïve (*p* = 0.046), NK cell activated (*p* = 0.019), macrophage M0 (*p* < 0.0001), and eosinophils (*p* = 0.0093) significantly increased in the high-risk group (Figure 5B). The stacked bar chart of the proportions of 22 TIICs in the patients from both groups is exhibited in Supplementary Figure S1B. Among all TIICs with significant differences in abundance, T-cell CD4 memory resting, macrophage M0, and T-cell CD8 accounted for the highest proportions, demonstrating a key immunological function of them in the development and progression of LUAD. In addition, the ESTIMATE results revealed that LUAD patients with low risk had a higher stromal score, immune score, and ESTIMATE score but lower tumor purity than those in the high-risk group (Figures 5C–F).

**FIGURE 6:**
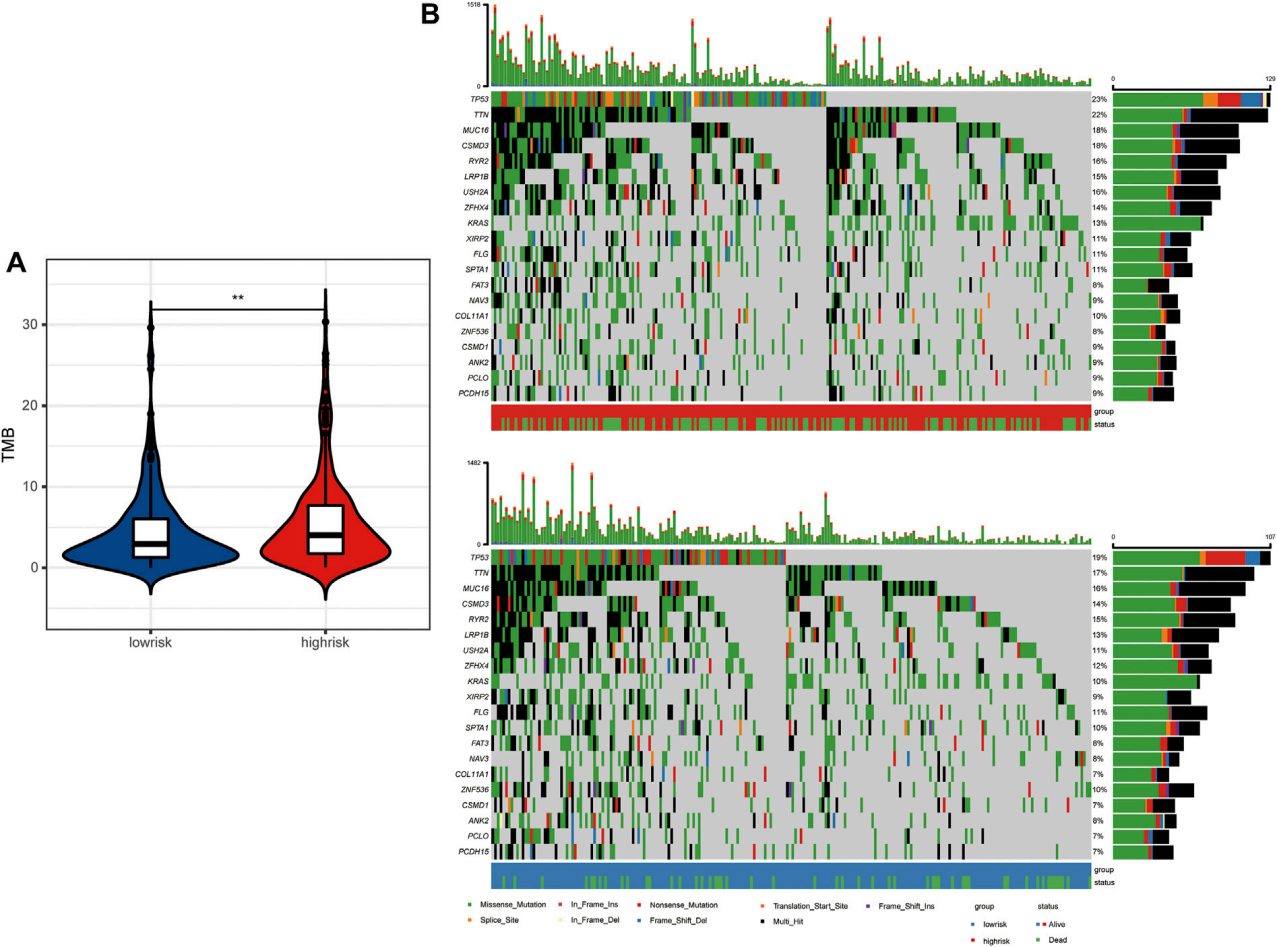
Tumor mutation landscape between high- and low-risk LUAD patients. **(A)** Tumor mutation burden between high- and low-risk groups. **(B)** Tumor mutation landscape of high- and low-risk groups.

Antigens produced by tumor mutations were closely related to immunotherapy. We found that the tumor mutation burden (TMB) of LUAD patients in the high-risk group was significantly higher than that in the low-risk group (Figure 6A). In detail, the mutation frequency of the top 20 genes in the high-risk group was higher than that in the low-risk group (Figure 6B). Moreover, TP53, TTN, MUC16, CSMD3, and RYR2 ranked as the top five genes with the highest mutation frequency in both groups. Among them, P53 mutations are mainly missense mutation and non-sense mutation, while TTN, MUC16, CSMD3, and RYR2 mutations are mainly missense mutation and multi-hit.

**FIGURE 7:**
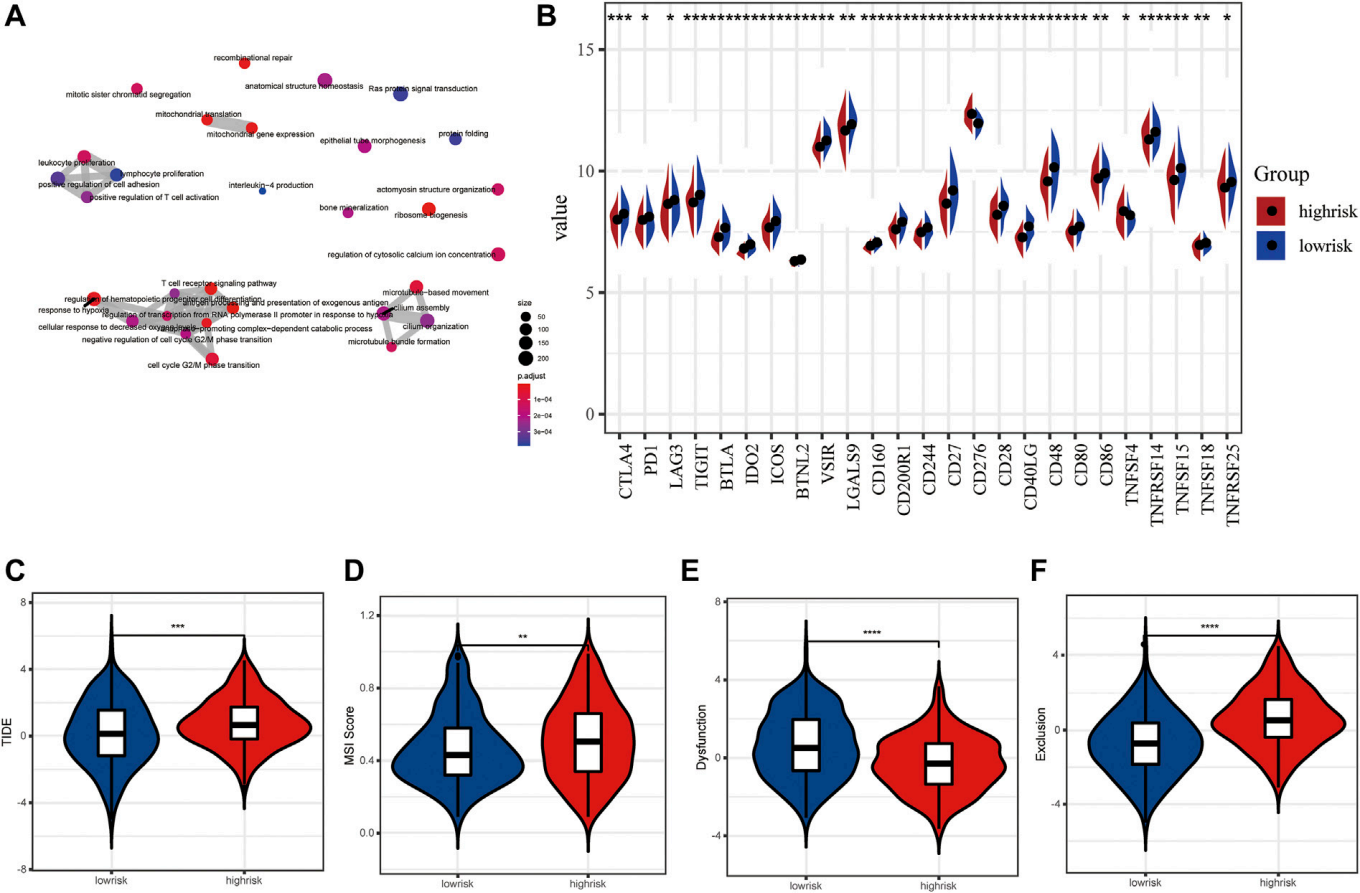
Immune checkpoint inhibitors and immunotherapy between high- and low-risk LUAD patients. **(A)** Tumor mutation burden between high- and low-risk groups. **(B)** Tumor mutation landscape of high- and low-risk groups. **(C–F)** TIDE score, MSI score, dysfunction score, and exclusion score between high- and low-risk groups.

The up-regulated genes were mainly enriched in the biological processes such as the ncRNA metabolic process, post-translational protein modification, and the ATP metabolic process (Supplementary Figure S1A), while the down-regulated genes were involved in the process of T-cell activation, regulation of T-cell activation, leukocyte proliferation, and regulation of lymphocyte-mediated immunity (Supplementary Figure S1B). Overall, all DEGs were related to several biological processes including the T-cell receptor signaling pathway, antigen processing and presentation of exogenous antigen, leukocyte proliferation, and response to hypoxia (Figure 7A). Based on these DEGs, GSEA and several dominantly enriched pathways, including immune-related pathways such as TNFα signaling via NF-κB, and other pathways including angiogenesis, epithelial–mesenchymal transition, estrogen response early, estrogen response late, glycolysis, and hypoxia were also performed (Supplementary Figure S1C).

As we all know, an immune checkpoint inhibitor is a promising lung cancer treatment strategy that has emerged in recent years. Compared with the high-risk group, 25 emerging immune checkpoints including PD1, CTLA4, and LAG3 were highly expressed, while CD276 and TNFRSF14 showed low expression level in the low-risk group (*p* < 0.05) (Figure 7B). Then, we utilized the tumor immune dysfunction and exclusion (TIDE) algorithm to predict the efficacy of response to immunotherapy. Interestingly, we found that the TIDE score of the low-risk group was significantly lower than that of the high-risk group, indicating more likely effectiveness for immunotherapy in the low-risk group (Figure 7C). In addition, the remarkably higher MSI score and T cell exclusion score and lower T cell dysfunction score were observed in the high-risk group (Figures 7D–F). These data further suggest that patients in the low-risk subtype may be more likely to benefit from immunotherapy.

### Nomogram Construction and Model Evaluation

**FIGURE 8:**
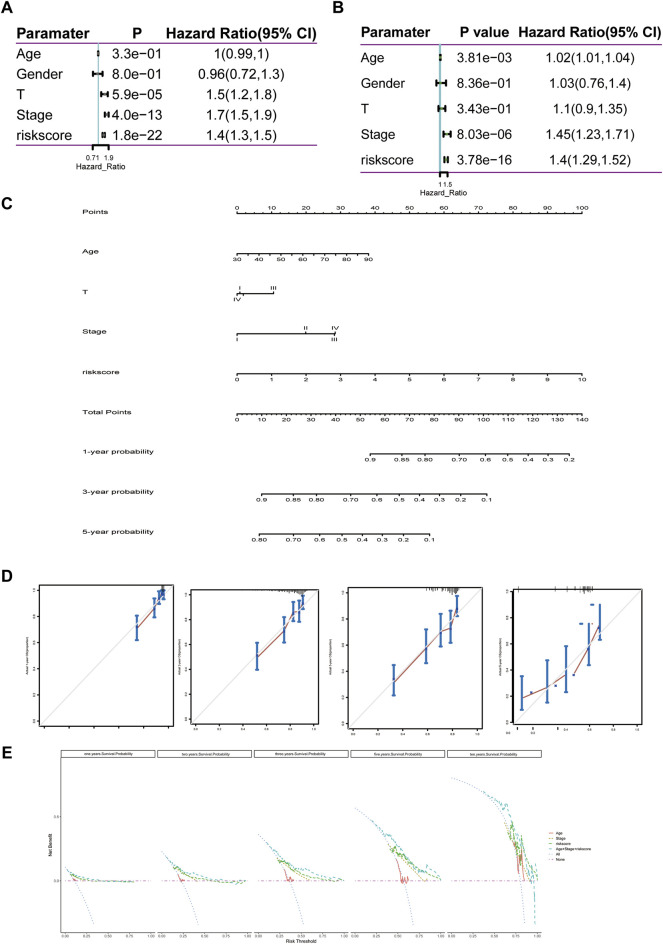
Construction and evaluation of the nomogram predicting the OS for TCGA-LUAD patients. **(A,B)** Univariate and multivariate regression analyses of clinical parameters. **(C)** Nomogram for predicting 1-, 3-, and 5-year OS of LUAD patients based on four independent prognostic factors. **(D)** One-, 2-, 3-, and 5-year calibration curves of TCGA-LUAD. **(E)** One-, 2-, 3-, 5-, and 10-year decision curve analysis of TCGA-LUAD. Comprehensive analysis of transcription, mutation, and copy number variation among 11 lipid metabolism-related genes.

The 11-LRG-based signature and main clinical factors were incorporated into univariate and multivariate Cox regression analyses to determine whether the signature could independently predict the survival of LUAD patients. First, univariate Cox analysis confirmed the T stage, tumor stage, and risk score of signature were associated with poor prognosis in patients with LUAD (Figure 8A). Then, multivariate Cox regression analysis further indicated that both the risk score of signature and tumor stage were significantly correlated with the OS of LUAD patients, which proved that the 11-LRG-based signature could independently predict the OS for the TCGA-LUAD patients (Figure 8B). Subsequently, a nomogram integrating the signature and tumor stage was constructed (Figure 8C). To evaluate the predictive performance of the nomogram, calibration curves and decision curve analysis (DCA) were conducted, which showed good performance in predicting the survival probability of LUAD patients. The results confirmed that calibration curves of 1-, 2-, 3-, and 5-year OS showed superior agreement between the predicted and actual survival LUAD cohorts (Figure 8D). Furthermore, DCA of the risk model showed the best net benefit for predicting 1-, 2-, 3-, 5-, and 10-year overall survival, especially for 5-year survival (Figure 8E). Thus, these results indicated that the nomogram was an optimal model in predicting the survival probability of LUAD patients.

**FIGURE 9:**
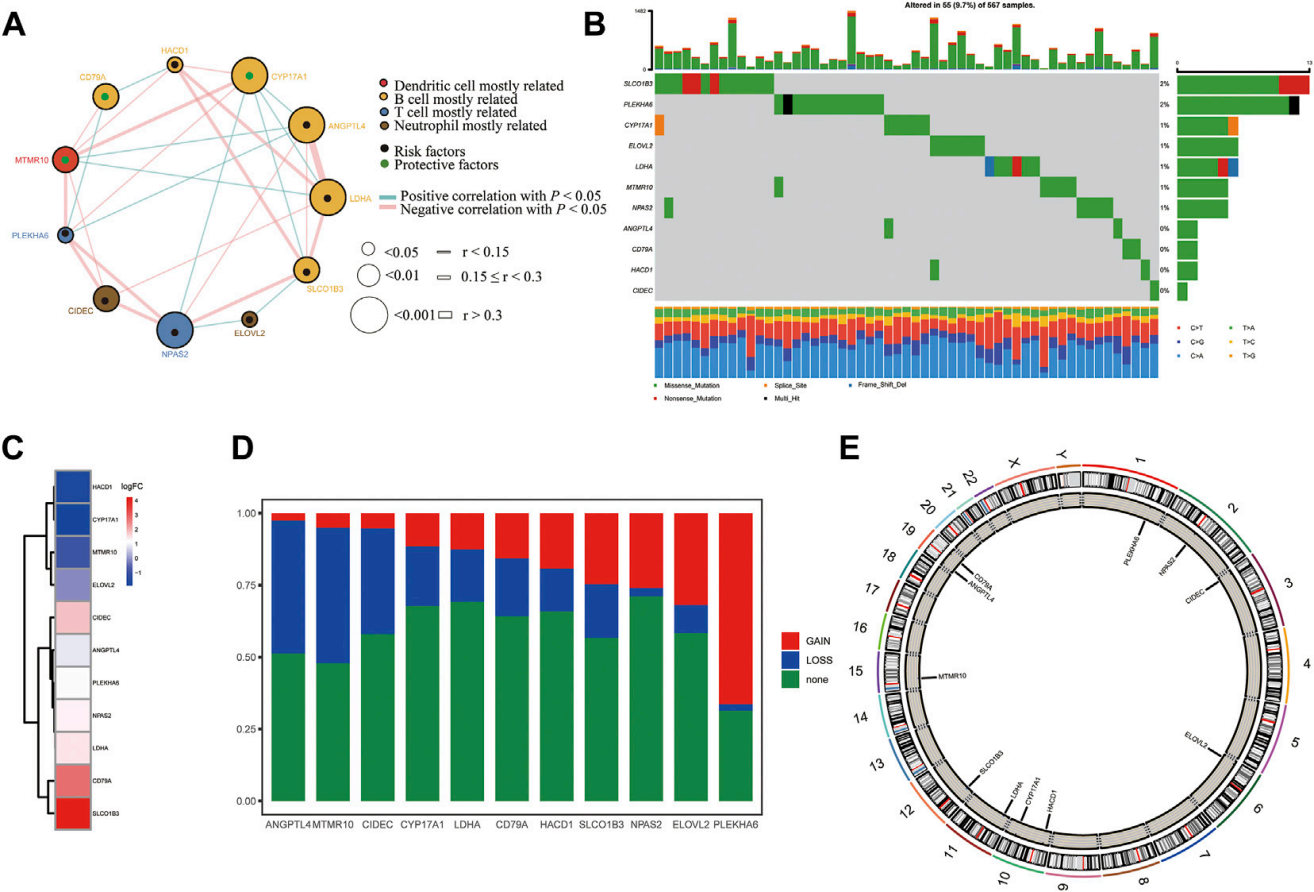
Landscape of the transcriptional regulation, incidence of copy number variations (CNVs), and somatic mutations of 11 hub lipid metabolism-related genes in LUAD. **(A)** Interaction between 11 hub genes in LUAD. The circle size represents the *p* value of each gene on the prognosis, and the range of values calculated by the log-rank test was *p* < 0.001, *p* < 0.01, and *p* < 0.05, respectively. Green dots in the circle mean risk factors of prognosis; black dots in the circle mean protective factors of prognosis. The lines linking genes show their interactions, and thickness shows the correlation strength calculated by the correlation coefficient was r < 0.15, 0.15 ≤ r < 0.3, and r > 0.3. Significant negative correlation is marked in blue, while significant positive correlation is marked in red. The gene associated with mostly related immune cells, such as dendritic cells, B cells, T cells, and neutrophils, based on TIMER is marked in red, yellow, blue, and brown, respectively. **(B)** Waterfall plot of tumor somatic mutation for 11 hub lipid metabolism-related genes. **(C)** Log (fold-change) of 11 hub lipid metabolism-related genes between 12 paired normal and LUAD tissues collected from Shanghai Tongren Hospital. **(D,E)** CNV alteration frequency and chromosome location of 11 hub lipid metabolism-related genes. CD79A with good prognosis was the molecule most relevant to the immune microenvironment.

We first summarized the transcriptional regulation, frequency of copy number variations (CNVs), and somatic mutations of 11 hub lipid metabolism-related genes in LUAD. The comprehensive landscape of 11 hub gene interactions, prognostic significance, and association with immune cells for LUAD patients was exhibited with the regulatory network (Figure 9A). We found that the six hubs including ANGPTL4, LDHA, CYP17A1, HACD1, SLCO1B3, and CD79A not only presented an extensive correlation in expression but also markedly correlated with B cells. RNA-seq data from 12 paired normal tissues and LUAD tissues collected from Tongren Hospital showed that SLCO1B3 (logFC = 4.23, *p* = 0.01), CD79A (logFC = 2.80, *p* = 0.00), LDHA (logFC = 1.41, *p* = 0.00), NPAS2 (logFC = 1.39, *p* = 0.01), PLEKHA6 (logFC = 1.23, *p* = 0.00), ANGPTL4 (logFC = 0.85, *p* = 0.04), and CIDEC (logFC = 1.82, *p* = 0.00) increased, while HACD1 (logFC = −1.67, *p* = 0.00), CYP17A1 (logFC = −1.91, *p* = 1.00), MTMR10 (logFC = −1.03, *p* = 0.08), and ELOVL2 (logFC = −0.16, *p* = 0.89) decreased in tumor (Figure 9C). Among 567 samples, 55 experienced mutations of 11 hub regulators, with frequency 9.7%. It was found that SLCO1B3 showed the highest mutation frequency followed by PLEKHA6 and CYP17A1, while ANGPTL4, CD79A, HACD1, and CIDEC did not show any mutations in LUAD samples (Figure 9B). The investigation of CNV alteration frequency showed several CNV alteration in 11 hub regulators, 5 of which including PLEKHA6, ELOVL2, NPAS2, SLCO1B3, and HACD1 focused on the amplification in copy number, while the rest including ANGPTL4, MTMR10, CIDEC, CYP17A1, LDHA, and CD79A had more popular frequency of CNV deletion (Figure 9D). In addition, the location of CNV alteration of 11 hub regulators on chromosomes was exhibited (Figure 9E).

**FIGURE 10:**
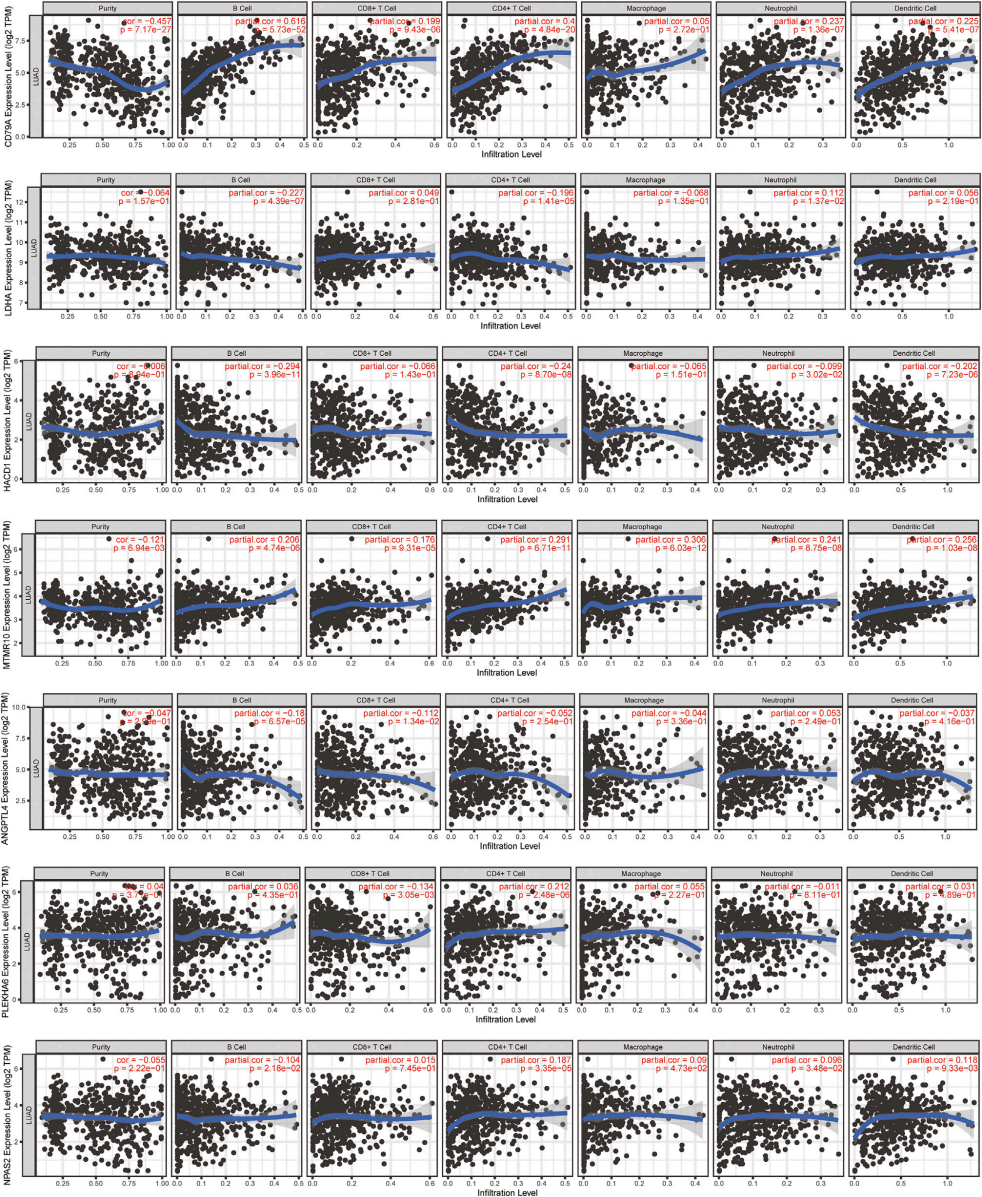
Relationship between 11 lipid metabolism-related genes and 6 kinds of immune cells using the TIMER database. The correlation scatter plot showed the relationship between 11 genes and infiltration of B cells, CD8^+^ T cells, CD4^+^ T cells, macrophages, neutrophils, and dendritic cells in LUAD.

In order to investigate the molecule most relevant to the immune microenvironment, we conducted the correlation analysis between 11 lipid metabolism-related genes and six kinds of immune cells using the TIMER database (Figure 10, Supplementary Figure S4). Among the 11 genes, we found that eight genes were closely related to the activation of B cells, four genes were related to the activation of CD8^+^ T cells, seven genes were related to the activation of CD4^+^ T cells, four genes were related to the activation of macrophages, seven genes were related to the activation of neutrophils, and four genes were related to the activation of dendritic cells (*p* < 0.05). Interestingly, CD79A showed a close relationship with B cells (r = 0.616, *p* = 5.73e-52), CD4^+^ T cells (r = 0.4, *p* = 4.84e-20), CD8^+^ T cells (r = 0.199, *p* = 9.43e-06), dendritic cells (r = 0.225, *p* = 5.41e-07), and neutrophils (r = 0.237, *p* = 1.36e-07), which may mediate the linkage between lipid metabolism and immunotherapy. Therefore, we further analyzed the expression, prognosis, and relationship with the risk score and immune microenvironment of CD79A in TCGA and TISIDB databases.

In the TCGA-LUAD cohort, the mRNA level of CD79A showed a negative correlation with risk score based on the 11-LMRG signature (r = −0.28, *p* = 3.8e-10), macrophage M0 (r = −0.21, *p* = 3.52e–06), and mast cell resting (r = -0.21, *p* = 2.32e–06), but a positive correlation with B-cell memory (r = 0.32, *p* = 5.34e–13), T-cell CD8 (r = 0.21, *p* = 1.44e–06), and plasma cells (r = 0.45, *p* = 2.52e–25) (Supplementary Figure S2A). Moreover, CD79A exhibited a strong correlation with several immune cells, immunoinhibitors, and immunostimulators (Supplementary Figure S2B), such as tumor-infiltrating lymphocytes including immature B cells (r = 0.697, *p* = 2.2–16) and activated B cells (r = 0.848, *p* = 2.2e–16), immunoinhibitors including BTLA (r = 0.659, *p* = 2.2–16) and TIGIT (r = 0.614, *p* = 2.2e–16), and immunostimulators including CD27 (r = 0.907, *p* = 2.2–16) and TNFRSF17 (r = 0.883, *p* = 2.2e–16) in the TISIDB database (Supplementary Figure S2C).

The mRNA expression level of CD79A was higher in LUAD tissues than in the non-tumor tissues in six datasets including GSE31210 (*p* = 3.4e-07), GSE75037 (*p* = 1e-05), GSE32683 (*p* = 0.00043), GSE11969 (*p* = 0.02), GSE30219 (*p* = 9.9e-06), and GSE81089 (*p* = 0.0093) (Supplementary Figure S3A), and it was negatively correlated with tumor stages (rho = −0.207, *p* = 2.59e-06) (Supplementary Figure S3C). In addition, the CD79A protein expression level increased in LUAD tissues than in the normal lung tissues in the HPA database (Supplementary Figure S3B). Survival analysis showed that the OS of the CD79A high expression group was significantly higher than that of the low expression group (*p* < 0.01) in the TISIDB database (Supplementary Figure S3D).

## Discussion

Lipid metabolism disorder is a hallmark of malignant tumor. Convincing evidence has suggested that abnormal metabolic activities could promote cancer development, due to its crucial role in angiogenesis, energy regulation, and cell proliferation (Liu et al., 2020a; Valença and Ferreira, 2020; Vazquez et al., 2020). Several lipid metabolism-based clinical prognostic models have been constructed for colorectal cancer, glioblastoma, and oral squamous cell carcinoma (Hu et al., 2019; Wu et al., 2019; Gharib et al., 2020). However, to our knowledge, the prognostic model of LMRG signature for LUAD patients has not been reported. In this case, our research focused on five lipid metabolism pathways strongly related to cancers, including fatty acid metabolism, glycerophospholipid metabolism, lipid raft, metabolism of lipids and lipoproteins, and phospholipid metabolism.

In this research, we attempted to construct an efficient prognostic model for LUAD patients based on 11 LMRGs screened by univariate Cox regression analysis, LASSO Cox regression analysis, and multivariate Cox stepwise regression analysis and calculated the risk score. Then, a nomogram was constructed to better predict accurate clinical outcomes. The nomogram integrated multiple prediction indicators based on multivariate regression analysis and then used line segments with scale to show the relationship between variables in the prediction model. A nomogram based on the autophagy gene signature had a concordance index of 0.71 to predict the survival possibility of NSCLC patients (Liu et al., 2019). Our nomogram generated a graphical statistical prediction model that assigns scores to each level of all independent factors, including age, T stage, and clinical stage and risk score. Calibration curves exhibited that the nomogram could accurately predict 1-, 2-, 3-, and 5-year survival probabilities. DCA curves showed that the nomogram had good benefit in predicting 1-year, 2-year, 3-year, 5-year, and 10-year survival, especially 5-year survival, meaning a potential translational value of the nomogram in LUAD patients.

Next, we conducted the lipid metabolism phenotype and differentially expressed gene analysis between high-risk and low-risk groups. Our results found up-regulated genes exhibited enrichment of post-translational protein modification, ATP metabolic progress, and ncRNA metabolic progress, whereas down-regulated genes mainly displayed enrichment of some immune progress, including regulation of T-cell activation, T-cell activation, and regulation of lymphocyte-mediated immunity, which hinted that different lipid metabolism status might be associated with immune microenvironment. A study found that cholesterol in the tumor environment can increase the CD36 expression of CD8^+^ T cells and then ingest too many fatty acids, leading to lipid oxidative damage and iron death, further resulting in the loss of its lethal function and promoting the growth of tumors (Bray et al., 2018). At present, it is believed that the relationship between lipids and immunity is that cancer cells use lipids to support their aggressive behavior and allow immune escape, while metabolic reprogramming of cancer cells destroys the balance between lipid synthesis and catabolism, resulting in lipid accumulation in the tumor microenvironment (Hanahan and Weinberg, 2011). Therefore, lipid metabolism and immunity are closely related in cancer, which is worthy of in-depth exploration.

Immune checkpoints have become a promising strategy for the treatment of many cancers. Whether the immune microenvironment and response to immunotherapy between high- and low-risk groups differed was further explored. CIBERSORT is a powerful tool to deconvolute the expression matrix of 22 human immune cell subtypes based on the principle of linear support vector regression, which has been widely used in plenty of oncology research studies (Huang et al., 2019; Liu et al., 2020b; Deng et al., 2020). Immune cell infiltration analysis found a distinctly different immune microenvironment especially the abundance of T cells and B cells between high- and low-risk groups. Interestingly, a large number of clinical cohort studies also showed that there was a positive correlation between B-cell infiltration and response to immunotherapy in a variety of different tumor types, emphasizing the important role of B cells in anti-tumor immunity (Helmink et al., 2020; Petitprez et al., 2020). T-cell failure has been identified as an important mechanism for cancer cells to escape host immunity (Marijt et al., 2019). Our results showed that, of all tumor-infiltrating immune cells (TIICs), the abundance of B-cell memory, T-cell CD8 memory, and T-cell CD4 memory resting significantly decreased in the high-risk group, which was consistent with the GO enrichment result that the immune process was inhibited, indicating there existed immune escape caused by T-cell depletion in the high-risk group. Higher tumor TIDE prediction scores were associated not only with poor efficacy of immune checkpoint inhibition therapy but also with poor survival of patients treated with anti-PD1 and anti-CTLA4 (Zarogoulidis et al., 2013). We then analyzed the expression of 25 common immune checkpoint molecules (including CTLA4, PD1, PD-L1, TIGIT, and LAG3). The results showed that 23 of 25 immune checkpoint molecules were lowly expressed in the high-risk group, suggesting the low-risk patients may be more likely to benefit from the updated immunotherapy. However, the lower TIDE score in the low-risk group suggested that the immune checkpoint blocking (ICB) therapy effect may be worse in the high-risk group, whose overall survival time may be shortened. In general, these data further support that the prognosis of low-risk subtypes is better, which may have a better prospect for immunotherapy.

Subsequently, we tried to explore the relationship between the genomic changes of 11 LMRGs and the susceptibility of LUAD. Further analysis confirmed that six genes (ANGPTL4, LDHA, CYP17A1, HACD1, SLCO1B3, and CD79A) related to B-cell activation were highly co-expressed, three of which (SLCO1B3, CYP17A1, and LDHA) had higher mutation frequency and higher levels of copy number variation, as well as relating to the pathogenesis of LUAD. Therefore, we concluded gene mutation and copy number variation may affect the susceptibility of LUAD patients genotyped by the risk score through influencing B-cell function.

To find the correlation between lipid metabolism and TME, we next conducted further analysis with TIMER and TISIDB databases. TISIDB is a kind of tumor immune-related repository, including 4,176 records from 2,530 publications and 988 genes related to anti-tumor immunity (Ru et al., 2019). CD79A (IgA), a part of BCR complex, was one of the specific antibodies on the surface of B cells (Müller-Winkler et al., 2021). However, when the antigen binds to the BCR complex receptor, CD79A is phosphorylated and internalized to activate B cells into plasma cells or memory B cells. At the same time, the number of lipid raft-related receptors increased, which participated in the regulation of lipid metabolism (Eleftheriadis et al., 2020). A study also confirmed that the increased expression of CD79A protein was associated with good prognosis of LUAD patients (Enfield et al., 2019). Moreover, the strong correlation between CD79A and risk score, B cells, T cells, TIGIT, BTLA, CD27, and TNFRSF17 revealed the crucial role of CD79A in regulating the tumor microenvironment (TME) in LUAD patients. High-risk genes in the model, including ANGPTL4, LDHA, and NPAS2, have been reported to promote angiogenesis, glycolysis, drug tolerance, the invasive and migratory potential, and cell cycle and apoptosis in lung cancer patients or cell lines (Galaup et al., 2006; Park et al., 2016; Gu et al., 2017; Li et al., 2018). CIDEC, CYP17A1, ELOVL2, and HACD1 were also associated with a variety of cancers including clear cell renal cell carcinoma, prostate cancer, neuroblastoma, and uveal melanoma and mainly participated in the metabolism of lipid storage droplets, promotion of *de novo* androgen biosynthesis, and regulation of fatty acid and inflammatory response (Yu et al., 2013; Xiao et al., 2018; Ding et al., 2019; Xu et al., 2019). The molecular function of PLEKHA6 was related to the hormonal receptor, thus leading to the poor prognosis of breast cancer (Aushev et al., 2019). Overall, we believed that CD79A may be the crucial molecule connecting the two mechanisms due to its dual role in regulation of lipid metabolism *via* participating in B-cell activation and have the potential to influence the response to immunotherapy in LUAD patients. Specifically, when B cells are stimulated by antigens to differentiate into plasma cells and memory B cells, frequent BCR signal transmission leads to the increase of lipid raft quantity and energy demand. At this time, lipid droplets act as regulators to maintain energy metabolism and provide material preparation (such as cholesterol and phospholipids) and energy for lipid rafts and their biological processes. However, once the lipid decomposition process is abnormal, lipid droplets would accumulate in the cells and further lead to lipid metabolism disorder and inflammation. Interestingly, CD79A may also play a role in enhancing the body’s immune capacity to repair lipid abnormalities and anti-inflammatory effect in this circumstance.

## Conclusion

In summary, our study constructed a high efficiency LMRG prognosis predictive model to separate LUAD patients into high- and low-risk groups, which may be helpful to promote the individualized immunotherapy for LUAD patients.

## Data Availability

The datasets presented in this study can be found in online repositories. The names of the repository/repositories and accession number(s) can be found in the article/Supplementary Material.

## Abbreviations

LUAD, lung adenocarcinoma; NSCLC, non-small-cell lung cancer; SCLC, small cell lung cancer; LMRGs, lipid metabolism-related genes; TCGA, The Cancer Genome Atlas; GEO, Gene Expression Omnibus; TIDE, tumor immune dysfunction and exclusion; TMB, tumor mutation burden; CNV, copy number variation; OS, overall survival; LASSO, least absolute shrinkage and selection operator; DEG, differentially expressed gene; PCA, principal component analysis; ssGSEA, single-sample gene set enrichment analysis; GSEA, gene set enrichment analysis; ROC, receiver-operating characteristic; AUC, area under the curve; ESTIMATE, Estimation of STromal and Immune cells in MAlignant Tumor tissues using Expression data; ICI, immune checkpoint inhibitor; DCA, decision curve analysis; TIMER, Tumor IMmune Estimation Resource; MSigDB, Molecular Signatures Database; FDR, false discovery rate; TIIC, tumor-infiltrating immune cell.
