# Supplementary material for: A Novel Signature of Lipid Metabolism-Related Gene Predicts Prognosis and Response to Immunotherapy in Lung Adenocarcinoma
Source: Front Cell Dev Biol. 2022 Feb 28;10:730132. doi: 10.3389/fcell.2022.730132 (PMC8918775; doi:10.3389/fcell.2022.730132)

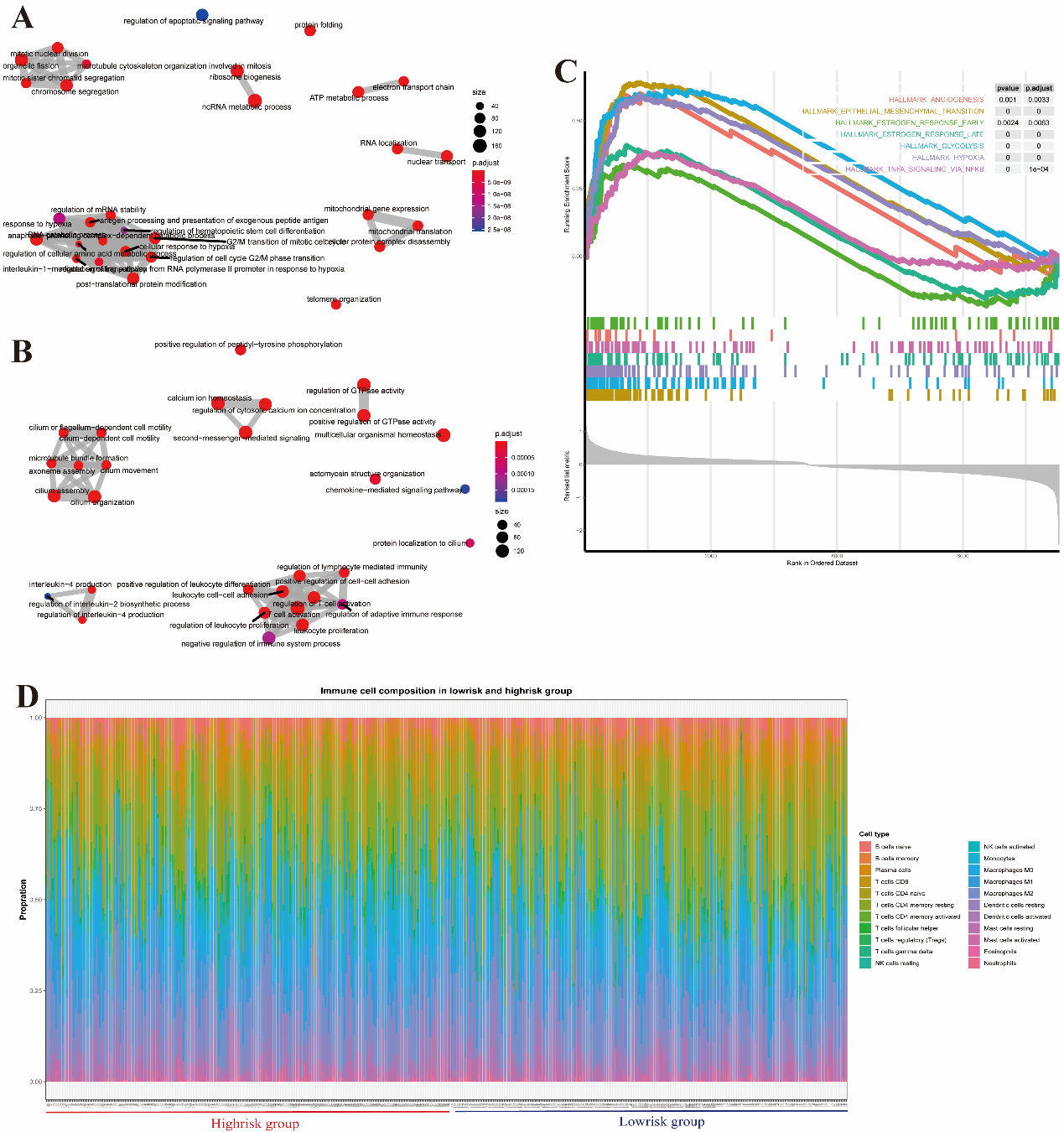


**Figure S1. Tumor immune microenvironment analysis between high and low risk group in TCGA-LUAD patients.** Gene Ontology (GO) enrichment map of up-regulated **(A)** and down-regulated **(B)** DEGs between high- and low-risk group. **(C)** GSEA of the DEGs between high- and low-risk group. (C) Grouped stacked bar chart of TIICs between high- and low-risk group. DEG: Differentially expressed genes; GSEA: Gene Set Enrichment Analysis; TIIC: Tumor infiltrating immune cell; ICI: immune checkpoint.


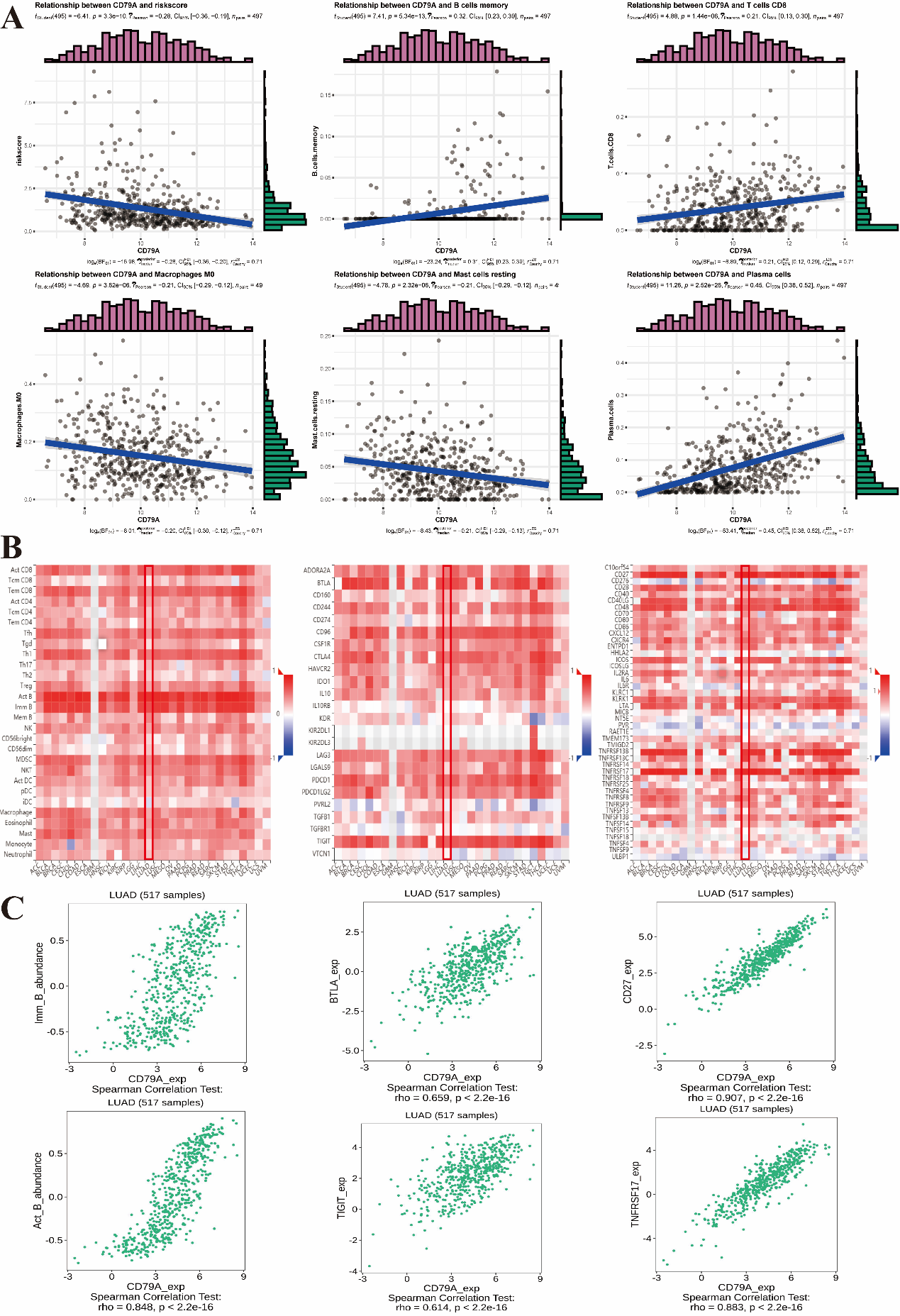


**Figure S2. The relationships between CD79A mRNA expression and immune cells and immunomodulators. (A)** Correlation between CD79A and riskscore, different immune cells in TCGA-LUAD cohort. **(B)** Heatmap of correlation between CD79A and immune cells, immunoinhibitors and immunostimulators in pan-cancers **(C)** Correlation between CD79A and the abundance of immature B cell and activated B cell, BTLA, TIGIT, CD27 and TNFRSF17 in TISIDB database.


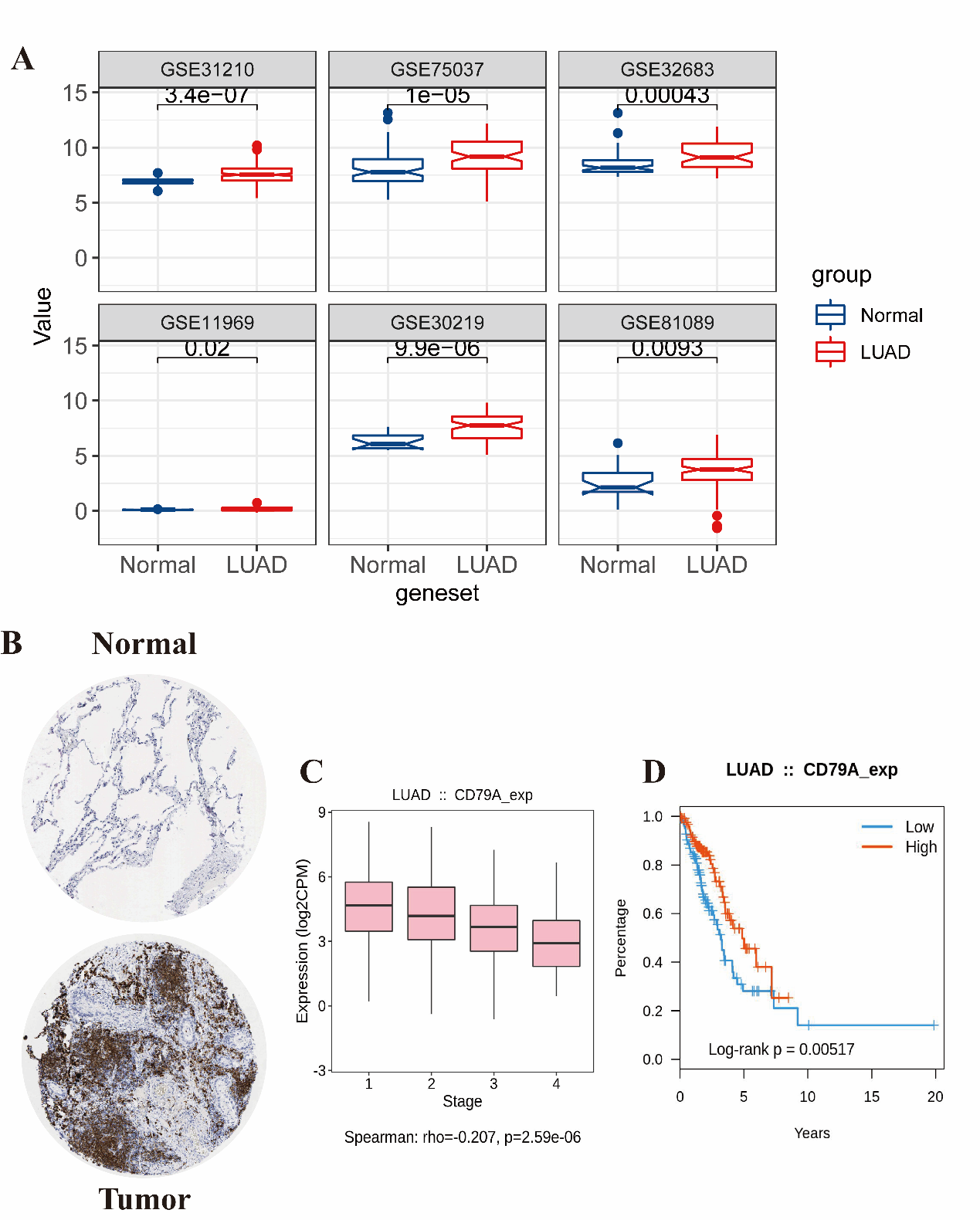


**Figure S3. High expression of CD79A is validated and associated with good prognosis for LUAD patients. (A)** Comparison of CD79A mRNA level between tumor and non-tumor group. **(B)** Comparison of CD79A protein level between tumor and non-tumor group in HPA database. **(C)** Correlation between CD79A and tumor stage for LUAD patients in TISIDB database. **(D)** Kapla-Meier survival curve estimating the OS probabilities for the low- versus high- CD79A expression group in TISIDB database.


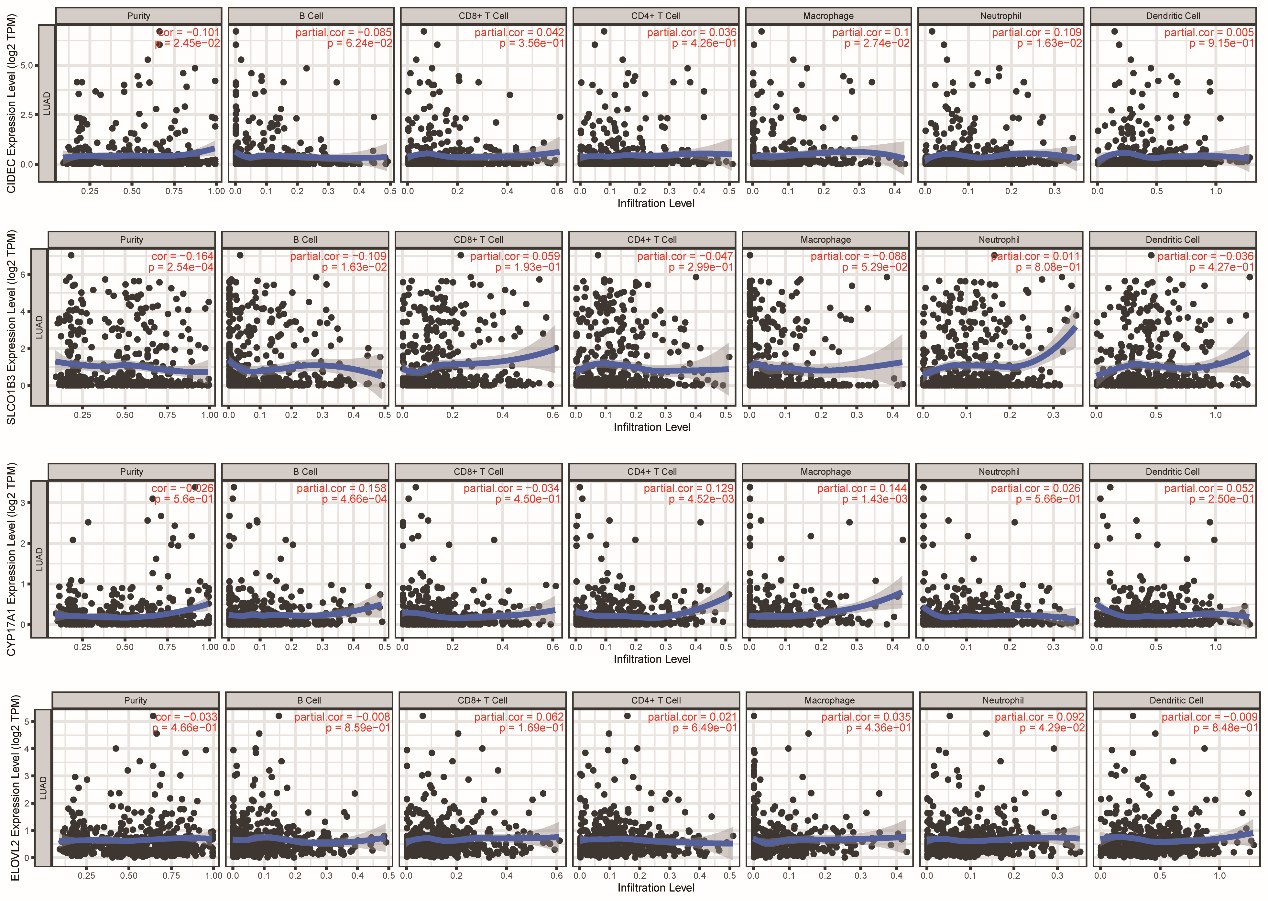

Supplement: Supplementary file 1 [file DataSheet1.docx]
